# Genotoxic Mechanism of Action of TBBPA, TBBPS and Selected Bromophenols in Human Peripheral Blood Mononuclear Cells

**DOI:** 10.3389/fimmu.2022.869741

**Published:** 2022-04-12

**Authors:** Anna Barańska, Agnieszka Woźniak, Katarzyna Mokra, Jaromir Michałowicz

**Affiliations:** Department of Biophysics of Environmental Pollution, Faculty of Biology and Environmental Protection, University of Lodz, Lodz, Poland

**Keywords:** tetrabromobisphenol A, tetrabromobisphenol S, pentabromophenol, Tribromophenol, DNA strand-breaks, DNA base oxidation, DNA adducts, peripheral blood mononuclear cells

## Abstract

Bromophenolic flame retardants (BFRs) are a large group of synthetic substances used in the industry in order to reduce the flammability of synthetic materials used in electrical and electronic devices, textiles, furniture and other everyday products. The presence of BFRs has been documented in the environment, food, drinking water, inhaled dust and the human body. Due to the widespread exposure of the general population to BFRs and insufficient knowledge on their toxic action, including genotoxic potential, we have compared the effect of tetrabromobisphenol A (TBBPA), tetrabromobisphenol S (TBBPS), 2,4,6,-tribromophenol (2,4,6-TBP) and pentabromophenol (PBP) on DNA damage in human peripheral blood mononuclear cells (PBMCs) (playing a crucial role in the immune system) as well as examined underlying mechanism of action of these substances. The cells were incubated for 24 h with studied compounds in the concentrations ranging from 0.01 to 10 µg/mL. The study has shown that examined BFRs induced single and, to a lesser extent, double strand-breaks formation and caused oxidative damage to pyrimidines, and particularly to purines in the incubated cells. PBMCs efficiently repaired the DNA strand-breaks induced by BFRs, but they were unable to remove completely damaged DNA (except cells treated with TBBPS). The greatest changes in the above-mentioned parameters were observed in cells incubated with TBBPA, while the smallest in PBMCs treated with TBBPS. The results have also revealed that tested compounds do not form adducts with DNA in PBMCs, while the observed changes were the most probably induced by indirect DNA-damaging agents, such as ROS and other reactive species.

## 1 Introduction

Bromophenolic flame retardants (BFRs) are ubiquitous chemicals widely used in the industry in the production of polymers, electrical and electronic equipment, textiles, furniture and other everyday products (1–3). Tetrabromobisphenol A (TBBPA) is the most widely used BFR. In 2004, it was estimated that the annual production of TBBPA in the United States, Japan and Israel reached approx. 170 thousand tones, which accounted for approx. 60% of the production of all brominated FRs worldwide (2). Due to massive production of TBBPA, this substance has been detected in the air, inhaled dust as well as in terrestrial and aquatic ecosystems (2, 4, 5). Tetrabromobisphenol S (TBBPS) was introduced into the market as TBBPA substitute. There is very limited data on the presence of TBBPS in the environment and its effect on living organisms. In the study of Wang et al. (6), TBBPS was detected in significant concentrations (up to 12.1 µg/L) in wastewater, while Ding et al. (7) observed that TBBPS altered the circadian rhythm network in the early life stages of zebrafish and potentially caused developmental delays in zebrafish embryos. Some studies have suggested that TBBPS may not be less toxic than TBBPA in humans. For instance, Liang et al. (8) observed that TBBPA and TBBPS had similar toxicity towards human embryonic stem cells. Both compounds disturbed neural ectoderm development, influenced axon growth and neuron transmission as well as dysregulated the WNT and AHR signaling pathways.

Brominated phenols like pentabromophenol (PBP), and particularly 2,4,6-tribromophenol (2,4,6-TBP) are widely represented in the environment and human surrounding. These substances have been repeatedly determined in the air, surface water, soil as well as home dust, food and drinking water (9). 2,4,6-TBP and PBP have also been shown to provoke various adverse effects in animals and human (3, 9, 10).

BFRs have been found in human plasma, placental tissue, adipose tissue, and breast milk samples (11, 12). TBBPA was detected in samples of human milk in the concentrations of 0.06 to 37.34 ng/g of fat and serum samples from both mothers and fetuses in similar range of concentrations (13). TBBPA was also found in plasma of Japanese men in a mean concentration of 950 pg/g fresh weight (14), while TBBPS was determined in a mean concentration of 0.593 μg/L of serum samples from pregnant women in China (15). Dufour et al. (16) determined 2,4,6-TBP in the concentrations from trace to 1.28 μg/L of blood of the general population of Belgium. In other studies, Feng et al. (17) detected 2,4,6-TBP in the concentrations of 5.57 ± 4.05 μg/L in the urine of the general population of China, while Gutierrez et al. (18) found very high mean concentration of 2,4,6-TBP in the urine of Chilean sawmills workers, which was 6.9 mg/g creatinine (approx. 6.9 mg per 1 L of urine).

The mechanism of bromophenolic FRs genotoxicity has not been elucidated. Moreover, literature data often offers conflicting information about the effects of these compounds on DNA. For instance, earlier studies have shown no genotoxic effects of TBBPA that could have been associated with the use of very low doses of this compound (19–21). However, more recent studies have indicated genotoxic potential of TBBPA in spermatozoa of mice (22), blood cells of spotted snake (*Channa punctatus*) (23) and mouse testicular cell co-culture model (24). 2,4,6-TBP was not genotoxic in *in vitro* bacterial tests (25, 26); however, it caused chromosomal aberrations (with and without metabolic activation) in *in vitro* tests on Chinese hamster cells (27). In case of other studied BFRs, data on their genotoxic effects is negligible. It was shown that PBP was not mutagenic in *Salmonella typhimurium* with or without metabolic activation (28), while genotoxic potential of TBBPS has not been studied.

Each cell under normal conditions is subjected to thousands attacks on its DNA each day (29), which may lead to genetic instability contributing to an increase in the rate of spontaneous mutations (30). Reactive oxygen species (ROS), and mostly hydroxyl radical (^•^OH) have been recognized as critical factors to the DNA damage, and our previous study showed that BFRs increased ROS, including ^•^OH levels in human PBMCs (31).

PBMCs play a key role in the body immune system. They are responsible for producing antibodies, killing virus-infected and cancerous cells, but also for regulating the immune system response (32). It has been proven that damage to PBMCs, and lymphocytes in particular (e.g. by xenobiotics) may contribute to the immune system dysfunction, which may result in autoimmune diseases (asthma, allergy) or cancer development (33, 34). Some studies have shown that BFRs may alter the immune system function. For instance, TBBPA has been shown to change tumor killing function of NK lymphocytes and alter secretion of various cytokines, including interferon gamma (IFNɣ), interleukin-1β (IL-1β) and tumor necrosis factor (TNF) (35). In another study, microarray analysis of uterine tissue of female Wistar Han rats showed that TBBPA downregulated genes in pathways of the immune response, which could lead to estrogen-mediated immunosuppression in tested animals (36).

Taking the above into consideration, we have decided to compare genotoxic effect of TBBPA, TBBPS, 2,4,6-TBP and PBP in human PBMCs, and examine underlying mechanism of action of these substances by evaluating single and double strand-breaks formation, purines and pyrimidines oxidation and DNA adducts creation in the tested cells.

## 2 Material and Methods

### 2.1 Chemicals

Tetrabromobisphenol A (99%, 2,2-bis(3,5-dibromo-4-hydroxyphenyl)propane) and pentabromophenol (98%, 2,3,4,5,6-pentabromophenol) were obtained from LGC Standards (Germany). Tribromophenol (pure ≤100%, 2,4,6-tribromophenol) was bought from Sigma-Aldrich (USA). Tetrabromobisphenol S (98.8%) was synthetized in the Institute of Industrial Organic Chemistry in Warsaw (Poland). Low melting point (LMP), normal melting point (NMP) agarose, fetal bovine serum (FBS) and DAPI (98%) were bought in Sigma-Aldrich (USA). Lymphocyte separation medium (LSM) (1.077 g/cm^3^) and RPMI 1640 with L-glutamine were purchased from Cytogen (Germany). Endonuclease III and human 8-oxoguanine DNA glycosylase were bought in New England BioLabs (USA). Potassium chloride (99.5%), sodium chloride (99.5%), sodium hydrogen carbonate (99%), ammonium chloride (99.5%), sodium wersenite (99.5%) and other chemicals were bought from POCH (Poland) and Roth (Germany).

### 2.2 Methods

#### 2.2.1 PBMCs Isolation and Treatment

PBMCs were isolated from the buffy coat (concentrated suspension of leucocytes and platelets) separated from whole blood in the Blood Bank in Lodz, Poland. Blood was collected from healthy, non-smoking volunteers (aged 18-40) showing no signs of infection disease symptoms. The method of PBMCs isolation was described in detail by Włuka et al. (31). The use of human blood in the study of the effect of tested BFRs on leucocytes was approved by the Bioethical Commission of Scientific Research at the University of Lodz (contract no. KBBN-UŁ/I/7/2011).

The cells were treated with tested compounds in the concentrations range from 0.01 to 10 µg/mL for 24 h at 37°C in 5% CO_2_ atmosphere in total darkness. The concentrations of examined BFRs corresponded to their levels determined in humans environmentally and occupationally exposed. Our previous study (31) proved that TBBPA, TBBPS, 2,4,6-TBP and PBP up to the concentration of 10 µg/mL did not decrease PBMCs viability below 80%. Cell viability (expressed in %) after treatment with TBBPA, TBBPS, 2,4,6-TBP and PBP at 10 µg/mL was 86.3 ± 3.55, 85.4 ± 1.98, 83.4 ± 2.47 and 81.2 ± 2.27, respectively (31). The analysis of cell viability was conducted using calcein-AM and propidium iodide stains. The samples were analyzed by means of flow cytometry.

The examined compounds were dissolved in DMSO. Final concentration of DMSO in untreated samples (negative control) and samples treated with TBBPA, TBBPS, 2,4,6-TBP or PBP was 0.2%. The above DMSO concentration was not toxic for PBMCs as assessed by all studied parameters.

All analyses of DNA damage included positive controls. The positive controls for alkaline and neutral comet assay were done based on previous experiments performed in our laboratory (37, 38).

Hydrogen peroxide at 20 µM was used in a positive control during analysis of single strand breaks (SSBs) formation and DNA bases oxidation (the cells were incubated with H_2_O_2_ for 15 min on ice). In order to induce double strand breaks (DSBs) formation, the samples were irradiated with 1.8 Gy/min for 5 min at room temperature.

#### 2.2.2 Comet Assay – Alkaline Version

A comet assay has been accepted as a simple, rapid and sensitive visual technique for assessing DNA damage. Alkaline version of comet assay can determine chemically or physically induced SSBs/DSBs and alkali labile sites, while neutral version of comet assay enables to determine selectively DSBs in the DNA of individual cells. In the comet assay, the cells are embedded in agarose on a microscope slide, and then are lysed with detergent and high salt to form nucleoids containing supercoiled loops of DNA linked to the nuclear matrix. After DNA staining, the release of DNA from a highly supercoiled DNA–protein complex is visually determined, which correlates with DNA damage detection (39, 40).

Alkaline version of the comet assay was carried out according to Singh et al. (40) with modifications (41), as described by Błasiak and Kowalik (42). A freshly prepared cells suspension in 0.75% LMP agarose dissolved in PBS was layered onto microscope slides, which was pre-coated with 0.5% NMP agarose. Then, the cells were lysed for 1 h at 4°C in a buffer containing 2.5 M NaCl, 0.1 M Na_2_EDTA, 10 mM Tris, 1% Triton X-100, pH 10. After cells lysis, the slides were placed in an electrophoresis unit. DNA was allowed to unwind for 20 min in the solution containing 300 mM NaOH and 1 mM Na_2_EDTA, pH > 13.

Electrophoretic separation was performed in the solution containing 30 mM NaOH and 1 mM EDTA, pH > 13 at ambient temperature of 4°C (the temperature of the running buffer did not exceed 12°C) for 20 min at an electric field strength of 0.73 V/cm (28 mA).

#### 2.2.3 Comet Assay - Neutral Version

A neutral version of the comet assay was used to assess DSBs formation (43). The electrophoresis was run in a buffer containing 100 mM Tris and 300 mM sodium acetate at pH 9.0 adjusted by glacial acetic acid. Electrophoresis was conducted for 60 min, after a 20 min equilibrium period, at electric field strength of 0.41 V/cm (50 mA) at 4°C.

#### 2.2.4 Oxidized Purines and Pyrimidines Detection (DNA Repair Enzyme Treatment)

Detection of oxidative DNA damage was conducted with the comet assay using endonuclease III (Endo III) and human 8-oxoguanine DNA glycosylase (hOGG1). The slides after cell lysis were washed three times (5 min, 4°C) in an enzyme buffer containing 40 mM HEPES–KOH, 0.1 M KCl, 0.5 mM EDTA, and 0.2 mg/mL bovine serum albumin, pH 8.0. Then, agarose on slides was covered with a volume of 50 μL of buffer containing 1 U of Endo III or hOGG1 or without the enzyme. Then, the slides were covered with cover glasses and incubated for 30 min at 37°C in a moist chamber. The cover glasses were removed and the slides were placed in an electrophoresis unit (44). DNA was allowed to unwind for 20 min in a solution containing 300 mM NaOH and 1 mM EDTA (pH > 13). The procedure was then conducted according to alkaline version of the comet assay.

We did not decide to calibrate the enzymes. According to New England BioLabs protocol, on which our experiment based on, dilution of hOGG1 and endoIII enzyme should be from 1: 102 to 1: 103 and from 1: 104 to 1: 105, respectively. It means that 50 μL of enzyme buffer with proper enzyme is equivalent of 0.08–0.8 U for hOOG1 and 0.05–0.5 U for endo III. Based on literature data (45) we decided to use 1 U of each enzyme per gel, which guaranteed their use in excess.

#### 2.2.5 DNA Repair

After 24 h of incubation, untreated cells (negative controls) and cells treated with TBBPA, TBBPS, 2,4,6-TBP or PBP at 10 µg/mL were washed and resuspended in RPMI 1640 medium with L-glutamine pre-heated to 37°C. Aliquots of the suspension were taken immediately (“time zero”) and 120 min later. In order to stop DNA repair, the samples were placed in an ice bath. DNA repair was assessed by the extent of residual DNA damage detection at time-point ‘0 min’ and ‘120 min’ using alkaline version of the comet assay.

#### 2.2.6 Comets Analysis

After electrophoresis, the slides were washed with deionized water, dried, stained with DAPI at 2 µg/mL and covered with cover slides. In order to prevent additional DNA damage, this procedure was carried out in limited light or darkness.

From each sample, 50 comets were randomly selected and the mean DNA value in the comet tail was taken as an indicator of DNA damage (expressed as a percentage). For one blood donor, two parallel tests with aliquots of the sample of the cells were performed for a total number of 100 comets. A total number of 300 comets (3 blood donors, n=3) was recorded to calculate mean ± SD.

The comets were observed at 200× magnification in an Eclipse fluorescence microscope AXIO SCOPE.A1 (Carl Zeiss, Germany) attached to Axiocam 305 color camera (Carl Zeiss, Germany) equipped with UV-1 filter block (an excitation filter of 359 nm and a barrier filter of 461 nm) and connected to a personal computer-based image analysis system Lucia-Comet v. 7.3 (Laboratory Imaging, Praha, Czech Republic).

#### 2.2.7 Plasmid Relaxation Assay

The plasmid relaxation assay was conducted to evaluate the effect of studied compounds on changes in DNA structure and their ability to form adducts with DNA. For this purpose, DNA plasmid from *E. coli* (pUC19) was used. Plasmid may be represented *via* various structural forms: super coiled (SC, completely intact DNA strands), linear (L, both DNA strands damaged) and open coiled (OC, damaged one of the DNA strands). During electrophoretic separation, the highest rate of migration is represented by super coiled form. Slower migration is shown by open coiled form, whereas the slowest rate is represented by linear form.

Plasmid pUC19 was incubated with tested compounds at 0.1 µg/mL, 1 µg/mL and 10 µg/mL. Negative control referred to a plasmid treated with DMSO (0.2%). A positive control was obtained by the exposure of the plasmid to a hydroxyl radicals (^•^OH) formed as a result of the Fenton reaction; ^•^OH induce DNA strand breaks formation, which lead to relaxation of supercoiled plasmid (observed as a DNA linear form – L). To initiate the Fenton reaction, a mixture of H_2_O_2_ at 200 µM and Fe^2+^ at 20 µM were added to the plasmid, which was incubated for 20 min at 37°C. After incubation, DNA gel loading buffer and Tris-EDTA buffer were added to the samples. Then, the samples were loaded onto 1% agarose gel and stained with ethidium bromide (0.5 µg/mL). Electrophoresis was performed in TrisAcetate-EDTA buffer for 60 min, at electric field strength of 5 V/cm (115 mA).

Gel was imaged in a Syngene Imagine Gels Documentation System under UV light and *via* Gel Documentation System Software Phoretix 1D. Image was saved as a TIFF file with a size of 16-bit. Then, image was evaluated using the Gel Analyzer tool of ImageJ, a public domain program from the National Institute of Health (NIH). Images were cropped from 1,280 x 1,020 pixels to 865 × 365 pixels to zoom into the gel. Before density analysis was done, background subtraction had been arranged. The profiles plot represents the average density value across a set of horizontal slices of each lane.

#### 2.2.8 Statistical Analysis

The tests by comet assay were carried out on blood from 3 donors. For each individual experiment (one blood donor), an experimental point was a mean value from 2 replications. Moreover, 3 experiments were conducted to assess DNA adducts formation. Data was expressed as mean value with standard deviation. The first step was to check data normality using the Shapiro-Wilk test. Statistical significance was examined on the basis of a comparison of averages using a one-way analysis of variance - ANOVA. In order to evaluate statistically significant differences between the tested samples, a multiple comparison test - the Tukey test (*post-hoc*) was used (46). The differences were considered to be statistically significant when p < 0.05. Analysis was performed using the STATISTICA 13 software (StatSoft, Inc, Tulusa, USA).

## 3 Results

### 3.1 DNA SSBs and DSBs Formation

**Figure 1:**
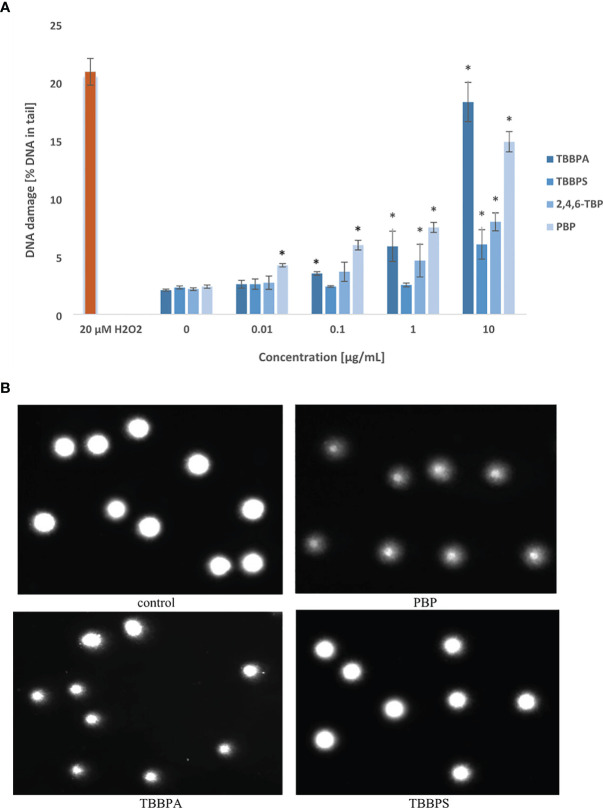
Total DNA strand breaks formation. **(A)** SSBs and DSBs formation in human PBMCs treated with TBBPA, TBBPS, 2,4,6-TBP and PBP at the concentrations of 0.01 µg/mL, 0.1 µg/mL, 1 µg/mL and 10 µg/mL for 24 h DNA damage was measured as the *percentage of DNA* in the comet *tail* using the alkaline version of the comet assay. Mean ± SD was calculated from 3 individual experiments (3 blood donors). Statistically different from negative control at *P<0.05. Statistical analysis was conducted using one-way ANOVA and a posteriori Tukey test. **(B)** Selected photographs of damaged DNA (comets) of human BPMCs incubated with DMSO at 0.2% (negative control) and tested BFRs at 1 µg/mL (comet assay, alkaline version). The photos were obtained using fluorescent microscope with 200x magnification.

The tested compounds induced SSBs/DSBs in DNA (**Figures 1A, B**). After 24-h of incubation the greatest changes were noted in cells treated with PBP, which even at 0.01 µg/mL caused DNA lesions. Much stronger DNA damage were noted in PBMCs incubated with PBP in the concentrations from 0.1 to 10 µg/mL. TBBPA at 0.1 µg/mL and 1 µg/mL also caused substantial damage to DNA, while at 10 µg/mL it caused greater DNA lesions than other tested BFRs. 2,4,6-TBP exhibited moderate genotoxic potential at 1 µg/mL and 10 µg/mL, while TBBPS only at 10 µg/mL induced relatively small DNA lesions (**Figure 1A**).

Selected photographs of damaged DNA (comets) of human PBMCs incubated with DMSO at 0.2% (negative control) and BFRs at 1 µg/mL were presented in **Figure 1B**.

**Figure 2:**
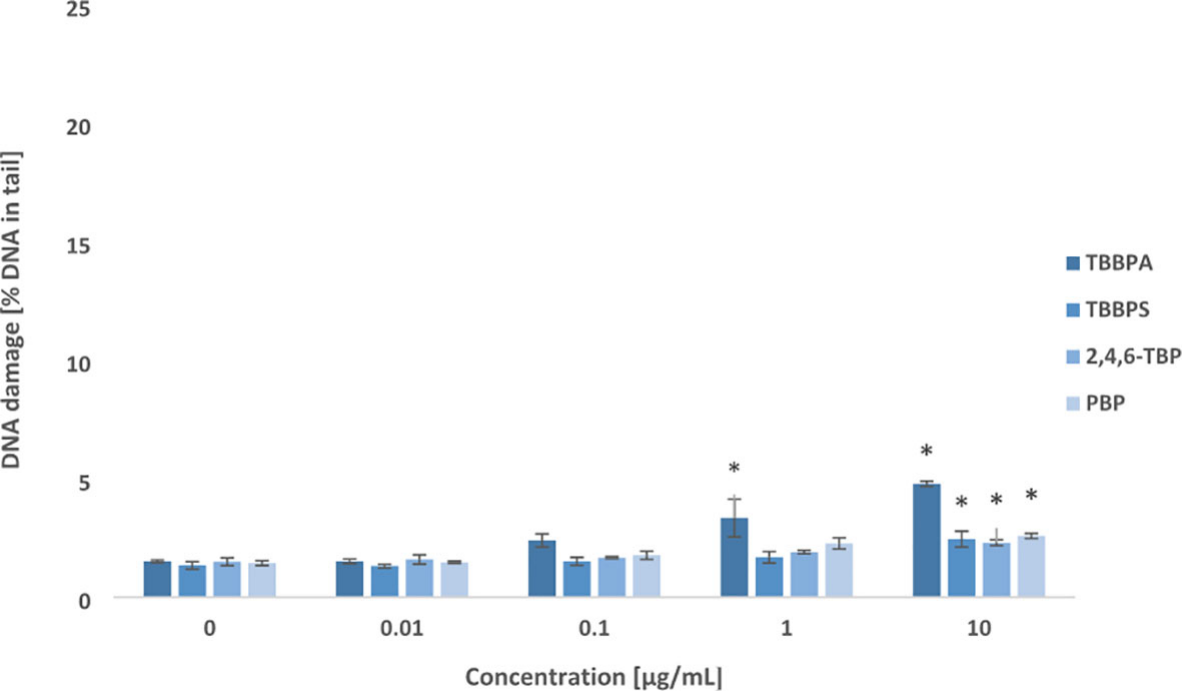
Double DNA strand breaks formation. DSBs formation in human PBMCs treated with TBBPA, TBBPS, 2,4,6-TBP and PBP at the concentrations of 0.01 µg/mL, 0.1 µg/mL, 1 µg/mL and 10 µg/mL for 24 h. DNA damage was measured as the *percentage of DNA* in the comet *tail* using the neutral version of the comet assay. Mean ± SD was calculated from 3 individual experiments (3 blood donors). Statistically different from negative control at *P<0.05. Statistical analysis was conducted using one-way ANOVA and a posteriori Tukey test.

After 24 h of incubation, all tested BFRs at their highest concentration of 10 µg/mL slightly increased DSBs levels in PBMCs. Among studied compounds, only TBBPA at lower concentration of 1 µg/mL was capable of inducing DNA DSBs formation in the incubated cells (**Figure 2**).

### 3.2 Oxidative Damage to DNA Bases

**Figure 3:**
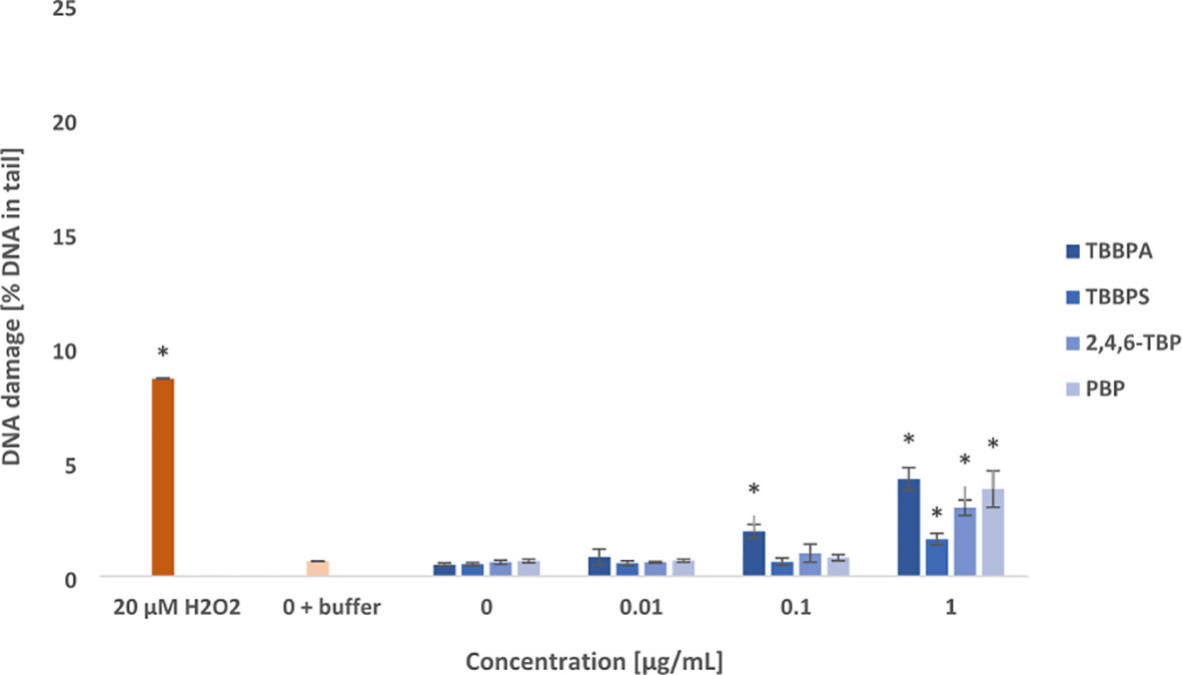
Oxidative damage to pyrimidines in DNA. Oxidative damage to DNA pyrimidines in human PBMCs treated with TBBPA, TBBPS, 2,4,6-TBP and PBP at the concentrations of 0.01 µg/mL, 0.1 µg/mL and 1 µg/mL for 24 h. DNA damage was measured as the *percentage of DNA* in the comet *tail* using the enzyme endo III and the alkaline version of the comet assay. The mean ± SD was calculated for 3 experiments (3 blood donors). Statistically different from negative control at *P<0.05. Statistical analysis was conducted using one-way ANOVA and a posteriori Tukey test.

**Figure 4:**
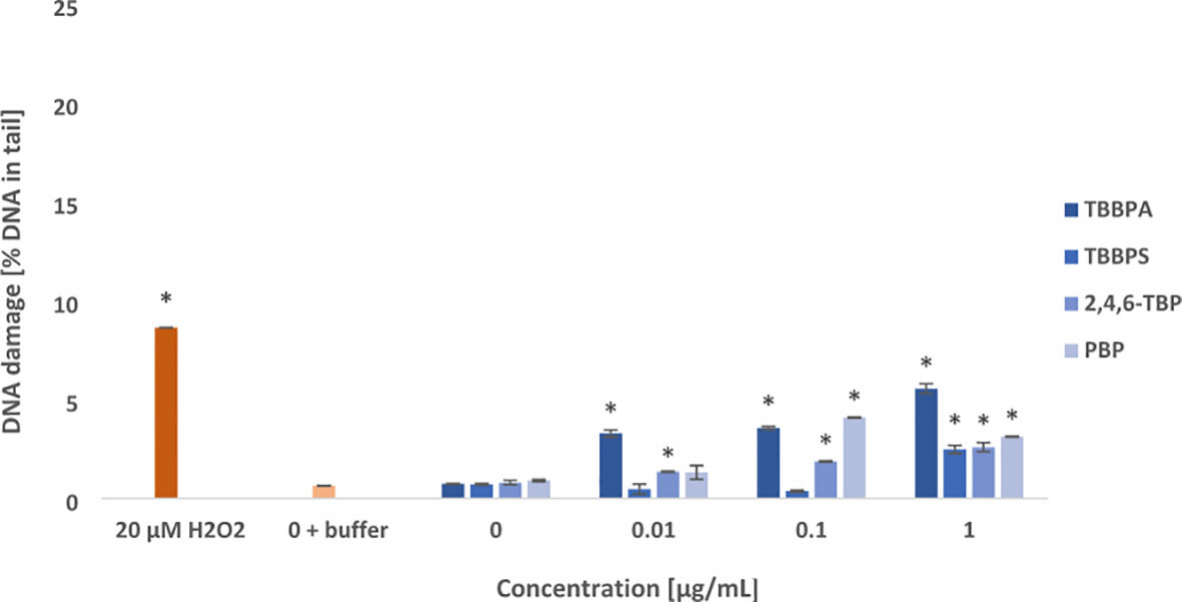
Oxidative damage to purines in DNA. Oxidative damage to DNA purines in human PBMCs treated with TBBPA, TBBPS, 2,4,6-TBP and PBP at the concentration of 0.01 µg/mL, 0.1 µg/mL and 1 µg/mL for 24 h. DNA damage was measured as the *percentage of DNA* in the comet *tail* using the enzyme hOGG1 and the alkaline version of the comet assay. The mean ± SD was calculated for 3 experiments (3 blood donors). Statistically different from negative control at *P<0.05. Statistical analysis was conducted using one-way ANOVA and a posteriori Tukey test.

Tested compounds after 24 h of incubation induced oxidative damage to pyrimidines and purines in PBMCs (**Figures 3**, **4**). Among studied BFRs, only TBBPA at 0.1 µg/mL caused slight increase in oxidized pyrimidines level, while all tested substances, and particularly TBBPA and PBP at highest concentration of 1 µg/mL induced oxidative damage to pyrimidines in the incubated cells (**Figure 3**).

It was observed that examined compounds caused greater damage to purines than pyrimidines in the PBMCs (**Figures 3**, **4**). TBBPA induced the greatest changes in the parameter examined increasing oxidized purines level even at 0.01 µg/mL, and more strongly at 0.1 µg/mL and 1 µg/mL. Similarly, 2,4,6-TBP in the concentrations range from 0.01 to 1 µg/mL was capable of provoking purines lesions, while PBP at 0.1 µg/mL and 1 µg/mL caused purines oxidation (**Figure 4**). The smallest changes were noted in cells treated with TBBPS, which only at 1 µg/mL caused small oxidative purine and pyrimidine oxidation in the studied cells (**Figures 3**, **4**).

### 3.3 DNA Repair

**Figure 5:**
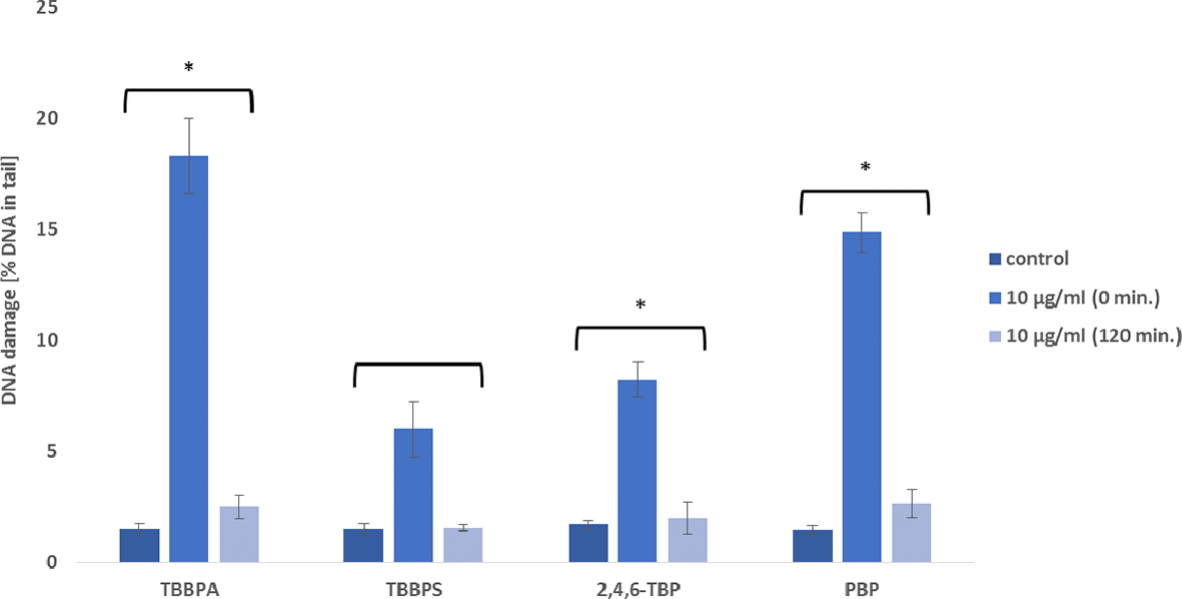
DNA repair capacity. Repair of damaged DNA in human PBMCs after 24 h of incubation with TBBPA, TBBPS, 2,4,6-TBP and PBP at 10 µg/mL. The repair was assessed after 120 min. of post incubation of the cells in medium deprived of these substances as a decrease in the extent of DNA damage (measured as the percentage of the DNA in comet tail) using the alkaline version of the comet assay. Mean ± SD was calculated from 3 individual experiments (3 blood donors). Statistically different from negative control at *P<0.05. Statistical analysis was conducted using one-way ANOVA and a posteriori Tukey test.

Tested compounds at the concentration of 10 µg/mL caused substantial SSBs/DSBs formation in PBMCs after 24 h of incubation (**Figures 1**, **5**). It was observed that PBMCs efficiently repaired DNA lesions, but they were unable to remove completely damaged DNA (except cells treated with TBBPS) after 120 min. post-incubation period (**Figure 5**).

### 3.4 Plasmid Relaxation Assay

**Figure 6:**
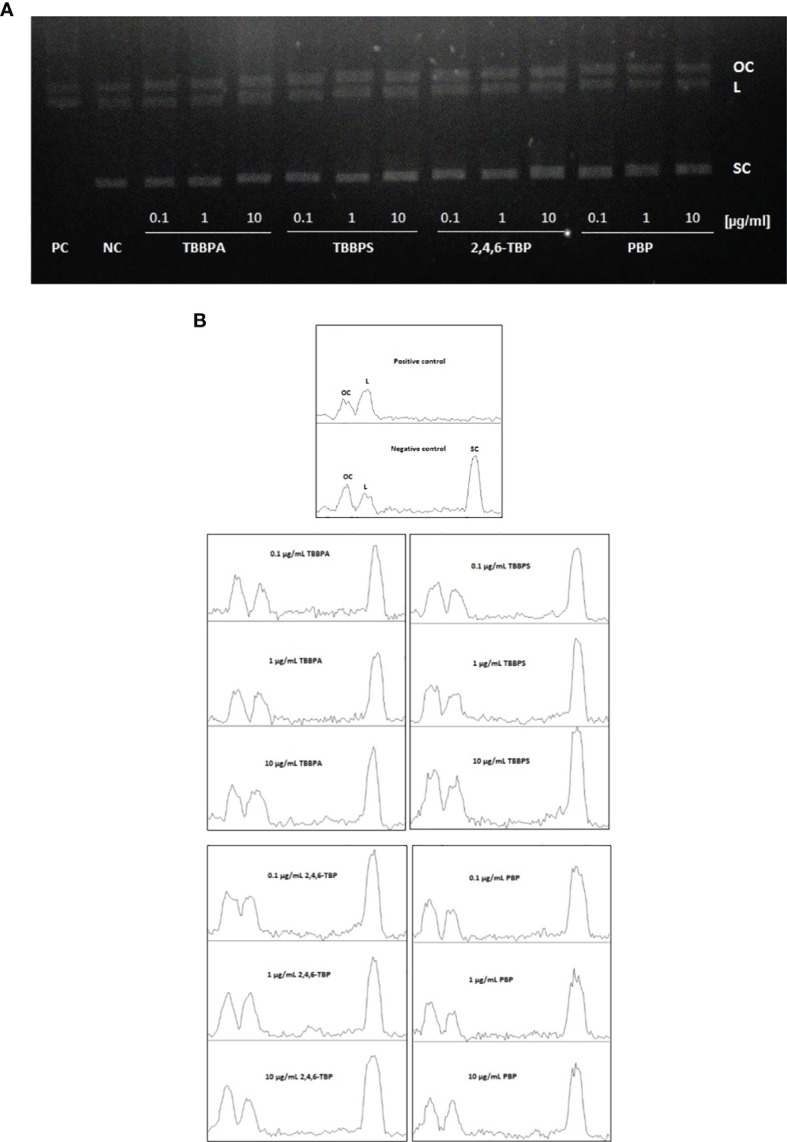
DNA adducts formation. Plasmid relaxation assay. **(A)** Plasmid DNA pUC19 was resolved on a 1% agarose gel, stained with ethidium bromide and visualized in UV light; line 1 - positive control (PC) (the plasmid was exposed to hydroxyl radicals generated in Fenton reaction), line 2 - negative control (NC) (pUC19 plasmid), lines 3-14 - pUC19 plasmid incubated with TBBPA, TBBPS, 2,4,6-TBP and PBP at 0.1 µg/mL, 1 µg/mL and 10 µg/mL. Structural differences between supercoiled (SC), open circular (OC) and linear (L) forms of the plasmid accounted for their different electrophoretic mobility. **(B)** Densitometric analysis of agarose gel was presented below the gel image. Open circular (OC) (as a consequence of DNA single strand breaks), linear (L) (as a consequence of DNA double strand-breaks) and supercoiled (SC) (undamaged DNA) forms of DNA plasmid are presented as peaks. Densitometric analysis was performed with the Gel Analyzer tool of ImageJ.

The results achieved during electrophoretic separation of pUC19 plasmid DNA revealed that neither brominated bisphenols nor bromophenols bound directly to DNA (**Figure 6A**). Similarly, densitometric analysis showed no changes in the amount of various plasmid forms after BFRs exposure, when compared to the negative control. That is why, it was concluded that tested compounds were incapable of creating adducts with DNA (**Figure 6B**).

## 4 Discussion

There are limited and inconsistent results concerning genotoxic potential of bromophenolic FRs (20, 23, 25, 27). Moreover, according to our best knowledge, no study has been conducted to describe genotoxic mechanism of action of these substances in any cell type or organism.

In this study, we have decided to assess genotoxic potential of TBBPA, TBBPS, 2,4,6-TBP and PBP in human PBMCs, which play a key role in protecting the body from pathogens and cancer cells as well as are involved in maintaining of body homeostasis (47).

The results have shown that some of examined compounds at relatively low concentrations (from 0.01 μg/mL) caused SSBs formation in PBMCs, while DSBs were detected in cells incubated with much higher concentrations (from 1 μg/mL) of tested substances (**Figures 1**, **2**). Among studied BFRs, TBBPA and PBP caused the greatest DNA damage.

Inconsistent literature results on TBBPA genotoxicity may be associated with usage of different research models, concentrations/doses or methods in evaluation of genotoxic potential of this substance. Earlier studies have shown no genotoxic potential of TBBPA (19–21), while more recent research works revealed DNA damaging potential of this substance. Yin et al. (24) determined an early DNA damage response marker γ-H2AX to assess the genotoxicity of TBBPA in mouse testicular cell co-culture model. They observed that after 24 h of incubation, TBBPA at 15 µM (8.1 µg/mL) caused significant increase of the number of γ-H2AX positive cells. Similarly, Liang et al. (48) observed that TBBPA at 25 µM (13.5 µg/mL) after 72 h of incubation increased the number of γ-H2AX positive mouse C18-4 spermatogonial cells. In another study, TBBPA at 20 µM (16.2 µg/mL) after 24 h of incubation caused DNA SSBs/DSBs formation in the IAR20 cell line (epithelial cells isolated from liver) (49). In *in vivo* study, Zatecka et al. (22) assessed DNA damage in spermatozoa of C57Bl/6J inbred mouse administrated with TBBPA at 200 µg/L dissolved in drinking water. Using terminal deoxynucleotidyl transferase-mediated dUTP nick end labelling (TUNEL), they detected significantly higher number of TUNEL-positive cells from TBBPA-treated animals. Similarly, Linhartova et al. (50) using comet assay showed that TBBPA in the concentrations range from 1.75 to 10 μg/L induced DNA fragmentation in spermatozoa nuclei of sterlet (*Acipenser ruthenus*), while Sharma et al. (23) observed that TBBPA at 5.09 µg/mL caused DNA damage in blood cells of snake head (*Channa punctatus*). Finally, TBBPA (0.2-0.8 µg/mL) has been shown to induce DNA SBs formation in gill and digestive gland cells of bivalve Farrer’s scallop (*Chlamys farreri*) (51).

There is almost no research on genotoxic potential of PBP. One study showed that PBP was not mutagenic in *Salmonella typhimurium* with or without metabolic activation (28). It is also worth noting that pentachlorophenol (PCP), which is a chlorinated analogue of PBP was capable of inducing significant increase in SBs formation in human peripheral blood lymphocytes (52).

This study showed that 2,4,6-TBP and particularly TBBPS induced lower level of SSBs/DSBs formation in comparison to other tested bromophenolic FRs (**Figures 1**, **2**).

Literature data does not provide any information on TBBPS genotoxicity; however Mokra et al. (53) revealed that its debrominated analogue bisphenol S (BPS) induced DNA SBs formation in human PBMCs.

Several studies have been conducted in order to assess 2,4,6-TBP genotoxicity, but no research work aimed to describe genotoxic mechanism of action of this substance. Analysis of 2,4,6-TBP mutagenicity in *Salmonella typhimurium* and *Escherichia coli* provided negative results (25, 54). Similarly, 2,4,6-TBP given intraperitoneally (75-300 mg/kg b.w.) to mice did not increase micronuclei formation in their bone marrow (54). Nevertheless, in most of eukaryotic models, 2,4,6-TBP has been shown to exhibit genotoxic potential. *In vitro*, in Chinese hamster lung cells (CHL/IU) 2,4,6-TPB in very high concentrations up to 1.6 mg/mL, induced chromosomal aberrations with and without metabolic activation (27, 54). Similarly, 2,4,6-TBP in high concentrations from 400 to 500 μg/mL was able to induce chromosomal aberrations in human peripheral blood lymphocytes both in the absence and the presence of metabolic activation (S9-mix) (20). More recently, *in vivo*, Lebaron et al. (55) using comet assay observed that 2,4,6-TBP mixed with bromoform and tribromoacetic acid induced DNA SBs in larvae of sea urchin (*Paracentrotus lividus*), while Heberle et al. (56) showed that 2,4,6-TBP significantly increased frequency of chromosomal aberrations in root cells of onion (*Allium cepa*). It was also reported that trichlorophenol, a chlorinated analog of TBP, caused DNA SSBs/DSBs formation in human lymphocytes (57).

The tested compounds caused oxidative damage to pyrimidines, and more strongly to purines (**Figures 3**, **4**). Generally, most of examined BFRs at lower concentrations (from 0.01 μg/mL) caused oxidative damage to DNA bases when compared with SSBs, and particularly DSBs induction. It was also observed that TBBPA caused the greatest oxidative damage to purines and pyrimidines, while TBBPS induced the lowest DNA bases lesions.

There is scarce data concerning oxidative DNA damage caused by BFRs. Choi et al. (58) observed that TBBPA given orally to Sprague-Dawley male rats strongly induced the production of oxidative DNA biomarker 8-hydroxy-2’-deoxyguanosine (8-OHdG) in the testis and kidney of the tested animals. In another study, 2,6-dibromohydroquinone, which is metabolite of TBBPA and 2,4,6-TBP at presence of Cu(II) caused 8-oxo-7,8-dihydro-2’-deoxyguanosine (8-oxodG) formation (59). Moreover, Michałowicz and Majsterek (60) observed that PCP and TCP were capable of inducing of oxidative DNA bases lesions in human peripheral blood lymphocytes, while Mokra et al. (37) showed that BPA and BPS induced pyrimidines and purines oxidation in human PBMCs.

DNA repair, including mismatch repair, the nucleotide excision repair or the base excision repair is responsible for the removal of DNA lesions (61, 62). Unrepaired DNA damage leads to a loss of genome integrity, and in the consequence increased risk of errors in the synthesis of both RNA and protein products. It has been shown that such increase of unrepaired lesions in DNA might be responsible for ageing process, cancer, atherosclerosis and degenerative diseases (63–66). For instance, inefficient oxidative DNA bases modifications by base excision repair (BER) may contribute to expansion of DNA trinucleotide repeat (TNR), which results in various neurodegenerative diseases development (67).

Our study has revealed that PBMCs efficiently repaired DNA lesions induced by tested BFRs, but they were not able to remove completely damaged DNA (except cells treated with TBBPS) (**Figure 5**). Similarly, Mokra et al. (50) assessed genotoxic potential of bisphenols in human PBMCs, and observed that tested cells completely removed DNA damage induced by BPS, but not by bisphenol A (BPA).

In order to elucidate the mechanism of the observed DNA damage, we explored the ability of tested BFRs to form DNA adducts. DNA adducts are created during interaction of physical factors and electrophilic chemical compounds with DNA (68). Using the conformation test, we evaluated the impact of examined compounds on the structure of DNA plasmid to find out whether DNA damage resulted from direct interaction between DNA and studied compounds.

The results have shown that none of tested compounds bound directly to DNA (created adducts) as no formation of linear structure of DNA plasmid was observed in any case (**Figures 6A, B**); therefore we suggested that DNA was damaged indirectly by ROS or/and other reactive species generated by tested BFRs.

ROS have been shown to be implicated in DNA damage, while hydroxyl radical (^•^OH) has the strongest ability to provoke oxidative DNA lesions (69). For instance, the creation of 8-hydroxylated purine in DNA is connected with addition of ^•^OH to the C8 of the purine base (70).

DNA-damaging effect of ROS in PBMCs treated with tested compounds is all the more likely because our previous study (31) showed that TBBPA, TBBPS, 2,4,6-TBP and PBP at very low (non-cytotoxic) concentrations (from 0.001 μg/L) were capable of generating total ROS and hydroxyl radical (at higher concentrations) in human PBMCs. Moreover the above study showed that PBP, and particularly TBBPA at 0.001 μg/L and 0.01 μg/L most strongly increased ROS level, which correlates with the results of this study showing that these substances exhibited the strongest genotoxic potential in the incubated cell. Similarly, Gao et al. (71) observed a correlation between SSBs/DSBs and 8-OHdG formation and an increase in ROS level in the SH-SY5Y cell line treated with brominated flame retardant PBDE-47 at non-cytotoxic concentration of 2.5 μg/L and 5 μg/L.

It must also be noted that TBBPA exhibited much stronger genotoxic potential than TBBPS in tested cells. Similar differences were observed by Mokra et al. (51) who showed that BPA caused stronger oxidative damage to DNA than BPS in human PBMCs. Taking the above findings into consideration, it may be suggested that sulphonyl group/methyl group(s) (but not bromine atoms) are mostly responsible for substantially different genotoxic effects provoked by TBBPA and TBBPS in tested cells.

It is worth noting that in physiological state, lymphocytes generate numerous DNA DSBs, which activate cellular DNA damage response (DDR). Interestingly, DDR capacity is different in various lymphocyte subsets being the strongest in NK cells, and the weakest in B lymphocytes, which correlates inversely with DNA damage-related survival (72). Recent studies have revealed that physiologic DNA SBs formation and DDR can initiate a genetic program that is unique and important for developing and maturation of lymphocytes. Nevertheless, elevated DNA DSBs formation that occur as a result of the exposure of lymphocytes to genotoxic agents may lead to improper activation of cell type-specific genetic programs, and thus disturb normal functions of lymphocytes (73). For instance, Innes and co-workers (74) observed that an increased DSBs formation in lymphocytes accelerated normal B cell maturation as well as induced a unique cancer-prone phenotype and the process that activated B cell response to antigen agent.

## Conclusions

(1) The results of this study have shown that bromophenolic FRs, such as TBBPA, TBBPS, 2,4,6-TBP and PBP caused SSBs, and to a much lesser extent DSBs formation in DNA of human PBMCs. (2) Tested compounds at low concentrations caused oxidative damage to purines, and to a lesser extent to pyrimidines. (3) The greatest changes in the above-mentioned parameters were observed in cells incubated with TBBPA, while the smallest in PBMCs treated with its commercial substitute TBBPS (4) PBMCs efficiently repaired DNA SBs induced by BFRs, but they were unable to remove completely damaged DNA (except cells treated with TBBPS). (5) It was revealed that tested compounds did not form adducts with DNA in PBMCs, while detected DNA lesions were the most probably induced by indirect DNA-damaging agents, such as ROS and other reactive species (6) Purines oxidation was induced by TBBPA and 2,4,6-TBP in the concentrations that were found in humans environmentally exposed to these substances, while all DNA damage types (excluding DSBs) occurred in PBMCs exposed to 2,4,6-TBP in the concentrations found in humans occupationally exposed to this compound.

## Data Availability Statement

The raw data supporting the conclusions of this article will be made available by the authors, without undue reservation.

## Ethics Statement

The use of human blood in the study of the effect of tested BFRs on leucocytes was approved by the Bioethical Commission of Scientific Research at the University of Lodz (contract no. KBBN-UŁ/I/7/2011). Written informed consent for participation was not required for this study in accordance with the national legislation and the institutional requirements.

## Author Contributions

AB analyzed the samples, interpreted the data and drafted the manuscript. AW analyzed the samples. KM prepared the samples and JM designed the study and revised the manuscript. All authors contributed to the article and approved the submitted version.

## Funding

This work was supported by statutory research (B2011000000191.01) admitted for Department of Biophysics of Environmental Pollution, Faculty of Biology and Environmental Protection, University of Lodz.

## Conflict of Interest

The authors declare that the research was conducted in the absence of any commercial or financial relationships that could be construed as a potential conflict of interest.

## Publisher’s Note

All claims expressed in this article are solely those of the authors and do not necessarily represent those of their affiliated organizations, or those of the publisher, the editors and the reviewers. Any product that may be evaluated in this article, or claim that may be made by its manufacturer, is not guaranteed or endorsed by the publisher.
